# Effect of gender on mortality and causes of death in cirrhotic patients with gastroesophageal varices. A retrospective study in Norway

**DOI:** 10.1371/journal.pone.0230263

**Published:** 2020-03-12

**Authors:** John Willy Haukeland, Milada Cvancarova Småstuen, Pia Pernille Pålsdatter, Moonisah Ismail, Zbigniew Konopski, Kristin Kaasen Jørgensen, Hans Lannerstedt, Håvard Midgard

**Affiliations:** 1 Department of Gastroenterology, Oslo University Hospital, Oslo, Norway; 2 Department of Public Health, Oslo Metropolitan University, Oslo, Norway; 3 Faculty of Medicine, University of Oslo, Oslo, Norway; 4 Department of Gastroenterology, Akershus University Hospital, Lørenskog, Oslo, Norway; 5 Department of Medicine, Lovisenberg Diaconal Hospital, Oslo, Norway; Chulalongkorn University, THAILAND

## Abstract

**Background & aims:**

The prognostic role of gender in patients with liver cirrhosis is not fully understood. Our primary aim was to assess how gender affects cumulative incidence and risk of death without liver transplantation (LT) in cirrhotic patients with gastroesophageal varices. Secondary aims were to assess the relationship between gender and cause specific death, risk of variceal bleeding and incidence rates of gastroesophageal varices in patients with cirrhosis.

**Methods:**

All new patients with gastroesophageal varices due to liver cirrhosis at Oslo University Hospital between 2006 and May 2016 were identified. Clinical data were retrieved retrospectively from hospital files. Causes of death were classified according to a specified protocol in cases of in-hospital-death, otherwise by data from the Norwegian Death Registry. Competing risk analyses were used to calculate cumulative incidences and risks of i) all-cause death, ii) cause-specific death and iii) variceal bleeding or re-bleeding.

**Results:**

Cumulative one- and five years incidence of death without LT in 266 included patients were 28% and 51%, respectively. In univariate analysis, risk of death was positively associated with age, Child Pugh class, alcoholic liver disease and presentation with variceal bleeding, and negatively associated with female sex. In a multivariate model, risk of death without LT was associated with female sex (SHR 0.59 [0.40–0.86]), age (SHR 1.05 [1.04–1.07] per year), Child Pugh class B (SHR 1.54 [1.03–2.32]) and Child Pugh class C (SHR 4.29 [2.57–7.17]). Variceal bleeding caused 27% of deaths. Adjusting for age and Child Pugh score, a trend towards reduced risk of death due to variceal bleeding was seen in women (SHR 0.53; [0.26–1.06]). High alcohol consumption was associated with increased risk of first variceal bleeding, both at univariate analysis (SHR 7.73 [1.71–34.9]) and multivariate analysis (SHR 13.9 [2.51–77.0]).

**Conclusions:**

Reduced mortality due to variceal bleeding may contribute to improved survival without LT in cirrhotic women with gastroesophageal varices.

## Introduction

Survival in cirrhosis is highly dependent on whether the patient is in a compensated or decompensated state; the latter defined by the presence of jaundice, encephalopathy, ascites or variceal bleeding [1]. Reflecting different aspects of decompensation, Child Pugh state is the most important prognostic factor in cirrhosis in addition to age [2]. The development of gastroesophageal varices in liver cirrhosis marks the transition to a more advanced disease state as it reflects the presence of clinical significant portal hypertension [3]. Most decompensated patients have clinical significant portal hypertension, however, it may also occur in compensated disease.

Although gender is found to be associated with survival in cirrhosis in some studies [1], its significance remains uncertain. Most studies indicating a gender effect report improved survival in women [1]. A recent study based on approximately 553 000 patients hospitalized with cirrhosis found in-hospital-mortality to be reduced by fourteen percent in females as compared to males [4]. It is not known, however, whether a positive effect of female sex is true for all cirrhotic patients, or whether it is restricted to decompensated patients. Moreover, none of the reports finding improved survival in women have explored possible relationships between survival and causes of death.

Many patients with cirrhosis die as a consequence of the liver disease, but determining the exact cause(s) of death may be a complex issue in this population. Studies on acute-on-chronic liver disease have emphasized the importance of a precipitating event, potentially triggering a downhill course with multi-organ failure and death [5]. Thus, in addition to rapid death due to exsanguination, variceal bleeding may also lead to death through ensuing complications. Understanding the root cause of death requires a comprehensive evaluation of the death process, since registered diagnoses in the hospital systems may not be sufficient for correct identification of the precipitating factor leading to death.

To improve our understanding of survival and causes of death among cirrhotic patients with gastroesophageal varices, in particular focusing on the role of gender, we conducted a retrospective study of all cases with gastroesophageal varices due to cirrhosis during a ten-year period at our hospital. The main objective was to explore factors associated with cumulative incidence and risk of death without liver transplantation (LT). Secondary aims were assessment of causes of death, predictors of variceal bleeding and incidence rates of gastroesophageal varices due to cirrhosis in a defined population.

## Material and methods

### Study population

**Fig 1:**
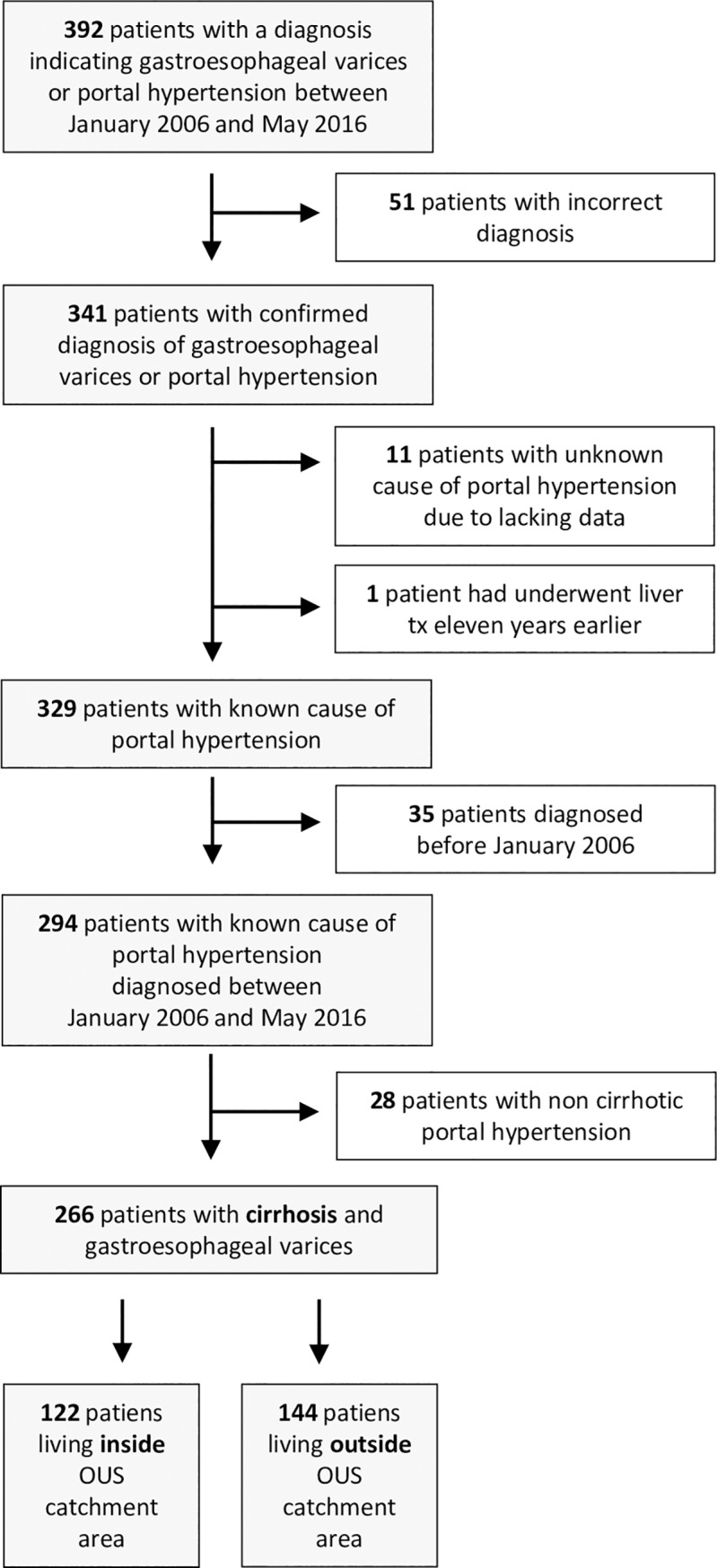
Flow chart describing identification of patients in the study.

Oslo University Hospital (OUS) is a tertiary centre treating both patients from the local catchment area (approximately 200 000 individuals) as well as patients referred from other hospitals in Norway, mainly other hospitals in Oslo and the South-Eastern part of Norway. We first identified 392 patients with a diagnosis of gastroesophageal varices (n = 391) or portal hypertension (n = 1) by searching the ICD10-codes K76.6, I85.0, I85.9 and I86.6 among all patients treated at the Department of Gastroenterology, OUS, between January 1^th^ 2006 and May 15^th^ 2016. Records of these patients were studied retrospectively. We excluded 51 patients in whom the diagnosis of gastroesophageal varices could not be verified, eleven cases were lack of data made it impossible to decide whether varices were caused by cirrhotic or non cirrhotic portal hypertension, one patient who underwent LT before inclusion in this study, 35 patients diagnosed before January 2006 and 28 patients with non-cirrhotic portal hypertension (Fig 1). Of the final study cohort of 266 patients, representing all new cases of gastroesophageal varices due to cirrhosis during the study period, 122 and 144 patients lived within and outside the catchment area of OUS, respectively. Date of inclusion was defined as the time of first verification of gastroesophageal varices by upper endoscopy. Clinical and biochemical data and dates of variceal bleeding, insertion of transjugular intrahepatic portosystemic shunt (TIPS), LT and death were collected from hospital files.

The study was performed as a quality assessment study approved by Department of Information Security and Data Protection, OUS, who waived the requirement for informed consent. All data were anonymized after collection from the hospital files. The Norwegian Directorate of Health recommended access to the Norwegian Cause of Death Registry for all deceased patients included in the study and gave permission to review hospital files at nine hospitals other than OUS to study causes of death (Lovisenberg Diaconal Hospital, Akershus University Hospital, Innlandet Hospital Trust, Vestre Viken Bærum Hospital, Diakonhjemmet Hospital, Østfold Hospital Trust, Vestfold Hospital Tønsberg, Ålesund Hospital, Nordland Hospital Bodø).

### Definition and etiology of cirrhosis

Cirrhosis was defined by histology in 97 patients and by a combination of clinical, biochemical, radiological and elastographic criteria in 169 patients. Causes of cirrhosis were registered as apparent from the records and categorized as alcoholic liver disease (ALD), chronic hepatitis B virus (HBV) infection, chronic hepatitis C virus (HCV) infection, non-alcoholic steatohepatitis (NASH), immunological liver disease (i.e. primary biliary cholangitis, primary sclerosing cholangitis, autoimmune hepatitis and overlap syndromes) and varia. Alcohol consumption was higher than five units per day for at least five years in patients with ALD, and less than two units per day in patients with NASH. Patients with chronic HBV or HCV infection and high alcohol consumption (more than two or three units per day for women and men, respectively) were grouped separately.

### Classification and treatment of gastroesophageal varices

Gastroesophageal varices were generally treated in accordance with international guidelines. Esophageal varices were either classified as small (deflatable) or large (non-deflatable). Non-selective beta-blockers (NSBB) or repeated endoscopic therapy was used for primary prophylaxis, and both strategies were used for secondary prophylaxis. In cases of variceal bleeding, patients were treated with vasoactive substances, antibiotics and endoscopic therapy within few hours after admission. Although endoscopic treatment and controls were done in a systematic manner, no reliable assessment of the use of NSBB could be obtained from the majority of the records. Fifteen patients were treated with TIPS, which was introduced at OUS in 2011. Variceal re-bleeding was defined as variceal bleeding that occurred more than three weeks after previous variceal bleeding.

### Causes of death

Causes of death could be assessed in 147 of 157 patients who died without LT during follow up. For individuals who died in hospital (n = 110) or shortly thereafter (n = 13), causes of death were determined by studying patient records using a specified protocol (S1 Table). In accordance with definitions suggested by EASL CLIF Consortium in the context of acute-on-chronic liver failure [5], biochemical criteria for liver failure were defined as either INR > 2.5 or serum bilirubin > 200 μmol/L. In the remaining 34 patients where death was not related to a hospital stay, cause of death could be defined in 24 patients by using data from the Norwegian Death Registry. Data were lacking or inconsistent in ten cases.

Liver related deaths were defined as either i) liver failure and infection, ii) liver failure and variceal bleeding, iii) liver failure and non variceal bleeding, iv) liver failure without variceal bleeding or infection, v) variceal bleeding without liver failure, and vi) advanced hepatocellular carcinoma (HCC). Non liver related deaths were classified as i) cancer other than HCC, ii) infection without liver failure, iii) cardiovascular disease, and iv) others.

### Statistical analyses

Crude differences between groups regarding continuous variables were assessed with Student’s t-test, ANOVA, Mann-Whitney U-test or Kruskal-Wallis test as appropriate. Differences in proportions were analysed using Chi-square test.

Cumulative incidence and risk of death were calculated using the Fine and Grey approach defining LT as a competing event [6]. Follow-up time was defined as time from date of inclusion to date of death, LT or end of follow-up on August 1^th^ 2018, whichever came first. Risks were expressed as subhazard rates (SHR). Univariate and multivariate analyses were performed to assess whether gender, age, Child Pugh state, etiology (ALD versus not ALD) or variceal bleeding at inclusion were associated with the risk of death. There was a significant interaction between gender and variceal bleeding at inclusion; the same analyses were therefore performed in patients with or without variceal bleeding at inclusion. We also performed separate analyses according to Child Pugh state (A versus B or C) to explore whether the effect of gender upon survival could be found in both compensated and decompensated disease.

Next, cumulative incidence and risk of LT were calculated using the Fine and Grey approach defining death as a competing event [6]. Risks were expressed as subhazard rates (SHR). Univariate analyses were performed to assess whether gender, etiology (ALD versus not ALD) or ethnicity (Caucasian versus non-Caucasian) were associated with the risk of LT. In a final analysis performed to assess the risk of LT according to etiology, the model was adjusted for gender and age.

To assess cumulative incidences and risks of specific causes of death without LT, we used the Fine and Grey approach. Ten patients with unknown cause of death were omitted from the analyses. Different models were fitted for i) death because of variceal bleeding, ii) liver related death other than variceal bleeding, iii) non liver related death and iv) death due to HCC. LT and other causes of death than the cause of interest were defined as competing events in each model. Gender, age and etiology were explored alone or in multivariate models. In the final model for risk of variceal death we adjusted for gender, age and Child Pugh class.

We assessed cumulative incidence and risk of first variceal bleeding among 151 patients with no bleeding at inclusion, and cumulative incidence and risk of first variceal re-bleeding among 115 patients with variceal bleeding at or prior to inclusion using the Fine and Grey approach [6] defining death as a competing event. For these analyses cases were censored at date of insertion of TIPS (n = 15), liver transplantation (n = 37) or at end of follow-up (i.e. December 31^th^ 2016 for patients living in OUS catchment area or possibly earlier for patients not living in OUS catchment area if follow up had been terminated), whichever came first. Univariate analyses were performed to assess whether age, gender, etiology, Child Pugh class, grade of esophageal varices and presence of gastric varices were associated with increased risks. A final model exploring the risk of first variceal bleeding was adjusted for gender, age, Child Pugh class, grade of esophageal varices and alcohol consumption.

Calculations of annual incidence rates of varices in the upper GI tract caused by liver cirrhosis were based on patients living in the OUS catchment area at time of diagnosis. Population data for this area and for the whole Norway were retrieved from Statistics Norway. Rates were calculated separately for age groups (0–19, 20–67 or >67 years) and gender for every year of the study period. The estimated sample rates were weighted with the relevant proportions from the population in Norway. As the incidence was higher among patients of African and Asian origin compared to the rest of inhabitants in Norway, calculations were done separately for these groups. Finally, the estimated incidence was weighted with the proportion of immigrants from Africa and Asia in Norway (0.05).

All tests were two-sided and p-values <0.05 were considered statistically significant. As our analyses were considered exploratory, no correction for multiple testing was performed. Analyses were performed using STATA v. 14.2. and SPSS v. 25.

## Results

### Baseline characteristics

Characteristics of the 266 patients included in the study are shown in Table 1. Notably, patients with viral hepatitis were younger compared to other etiologies, and the proportion of males was low in patients with immunological liver disease (14%) and high in patients with high alcohol consumption (79–83%). Most patients had advanced cirrhosis, classified as Child Pugh A, B and C in 31%, 41% and 28% of cases, respectively. Patients with high alcohol consumption had higher Child Pugh score and presented more often with variceal bleeding. Of all patients, 59% had large esophageal varices, and 20% had gastric varices, with no differences between various etiologies.

**Table 1:** Demographic, clinical and biochemical variables according to cause of cirrhosis in 266 patients with gastroeosophageal varices.

	All (n = 266)	ALD (n = 125)	Viral hepatitis + high alcohol # (n = 24)	Viral hepatitis + low alcohol ## (n = 41)	NASH (n = 15)	Immunological liver disease (n = 36)	Varia ### (n = 25)	*p-value*
**Age (SD), years**	59.1 (13.0)	60.0 (9.2)	53.8 (6.1)	52.5 (12.1)	64.8 (12.0)	61.5 (16.0)	63.1 (23.2)	0.001
**Male proportion (%)**	63.9	79.2	83.3	61.0	53.3	13.9	56.0	<0.001
**INR (SD)**	1.6 (0.5)	1.7 (0.6)	1.7 (0.4)	1.4 (0.3)	1.5 (0.4)	1.4 (0.4)	1.4 (0.2)	0.001
**Bilirubin (IQR), μmol/l**	28 (36)	37 (56)	43 (56)	20 (13)	26 (25)	28 (50)	18 (15)	<0.001
**Platelets (SD), x 10^9^/L**	119 (62)	122 (55)	98 (65)	93 (43)	112 (55)	155 (79)	123 (73)	<0.001
**AST/ALT-ratio (IQR)**	1.8 (1.0)	2.2 (1.0)	2.1 (0.9)	1.2 (0.7)	1.8 (0.5)	1.4 (0.8)	1.5 (0.5)	<0.001
**Child-Pugh**								<0.001
• **A(%)**	31.2	16.0	25.0	61.0	33.3	38.9	52.0	
• **B(%)**	40.6	45.6	29.2	26.8	46.7	41.7	44.0	
• **C(%)**	28.2	38.4	45.8	12.2	20.0	19.4	4.0	
**Variceal bleeding at inclusion (%)**	39.5	47.2	58.3	17.1	40.0	30.6	32.0	0.004
**Esophageal varices at inclusion**								0.174
• **None**	4.5	8.0	0.0	0.0	0.0	0.0	8.0	
• **Small (%)**	36.1	37.6	25.0	39.0	26.7	44.4	28.0	
• **Large (%)**	59.4	54.4	75.0	61.0	73.3	55.6	64.0	
**Gastric varices at inclusion**	19.5	20.0	20.8	19.5	20.0	11.1	28.0	0.381

#: HCV (n = 23), HBV (n = 1).

##: HCV (n = 33), HBV (n = 10), HIV (n = 3)

###: Cryptogenic liver cirrhosis (n = 17), cystic fibrosis (n = 2), hemochromatosis (n = 1), Wilson disease (n = 1), cardiac cirrhosis (n = 1), chronic Budd Chiari syndrome (n = 1), schistosomiasis (n = 1) and HIV related (n = 1).

Comparisons between men and women revealed several significant differences (Table 2). Importantly, while ALD was less frequent among women, immunological liver diseases were more common. Moreover, women were older and presented with slightly lower Child Pugh score compared to men.

**Table 2:** Demographic, clinical and biochemical variables according to gender in 266 patients with gastroeosophageal varices.

	Men (n = 170)	Women (n = 96)	*p-value*
**Age (SD), years**	57.6 (12.5)	61.6 (13.4)	0.016
**Cause of liver cirrhosis**			<0.001
• **ALD (%)**	57.6	28.1	
• **Viral hepatitis (%)**	26.5	20.8	
• **NASH (%)**	4.7	7.3	
• **Immunological liver disease (%)**	2.9	32.2	
• **Varia (%)**	8.2	11.5	
**INR (SD)**	1.6	1.4	0.004
**Bilirubin (IQR), μmol/l**	31 (41)	26 (23)	0.058
**Platelets (SD), x 10^9^/L**	109 (52)	137 (73)	<0.001
**AST/ALT-ratio (IQR)**	2.2 (1.1)	1.8 (0.7)	<0.001
**Child-Pugh**			0.065
• **A(%)**	27.1	38.5	
• **B(%)**	40.6	40.6	
• **C(%)**	32.4	20.8	
**Variceal bleeding at inclusion (%)**	42.2	34.4	0.201
**Esophageal varices at inclusion**			0.818
• **None**	4.7	4.2	
• **Small (%)**	34.7	38.5	
• **Large (%)**	60.6	57.3	
**Gastric varices at inclusion**	20.6	17.7	0.229

### Cumulative incidences and risk of death without LT

**Fig 2:**
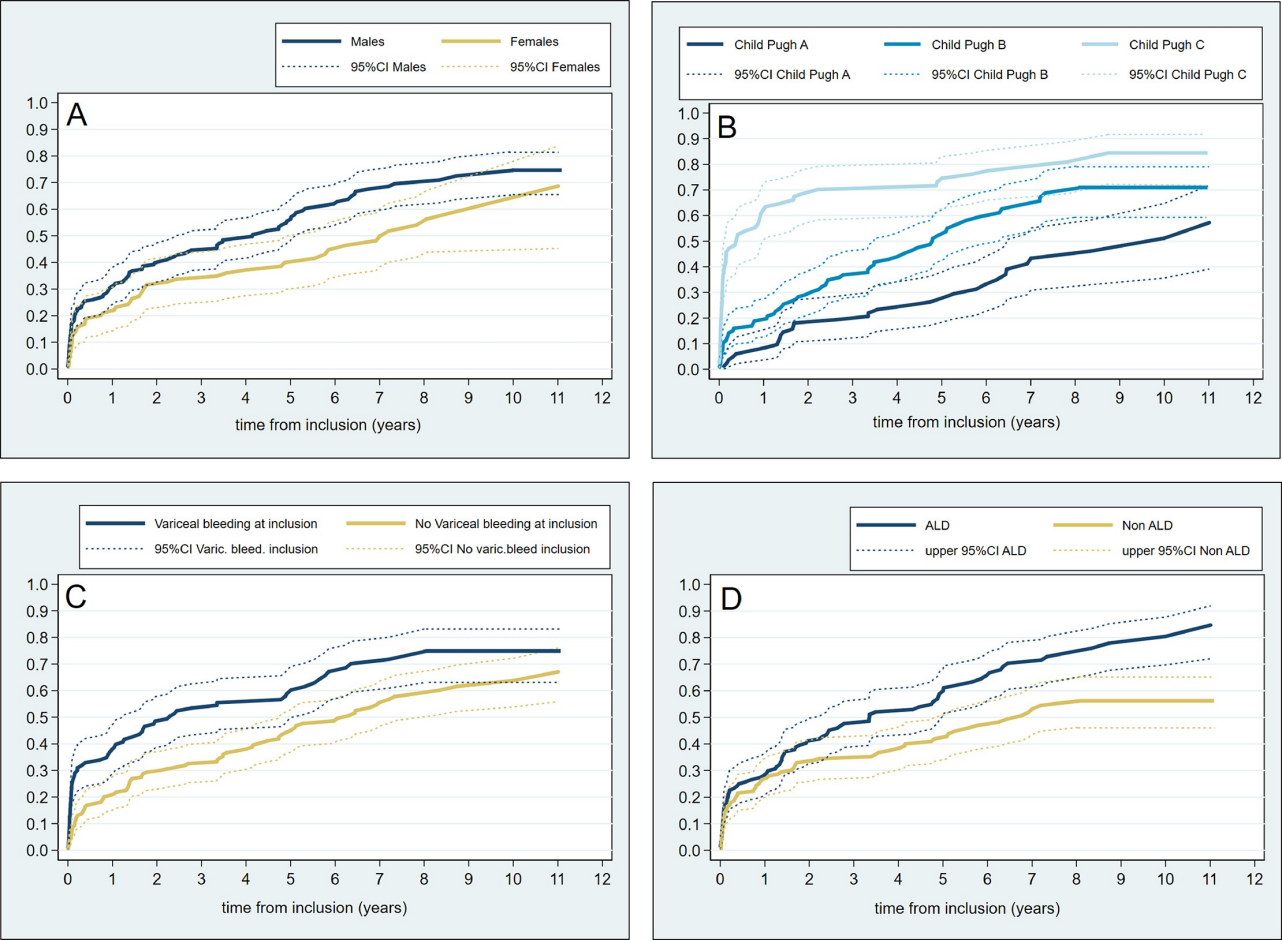
Cumulative incidences of death without LT in 266 patients with gastroesophageal varices. The incidence curves were constructed with the Fine and Grey approach treating LT as competing events. Death according to (A) gender, (B) Child Pugh class, (C) variceal bleeding at inclusion and (D) etiology.

During a median follow-up of 2.5 years (IQR: 0.4–5.3 years), the one- and five years cumulative incidence of death without LT were 0.28 (95% CI [0.22–0.34]) and 0.51 (95% CI [0.44–0.57]), respectively. Cumulative incidences of death without LT according to gender, Child Pugh class, etiology (ALD versus non ALD) and variceal bleeding at inclusion are shown in Fig 2. These factors, in addition to age, were all significantly associated with risk of death without LT at univariate analyses (Table 3).

**Table 3:** Univariate and multivariate competing risk regression to explore factors associated with risk of death without LT in 266 patients with liver cirrhosis and gastroesophageal varices.

	Univariate		Multivariate	
	SHR (95% CI)	p-value*	SHR (95% CI)	p-value*
Female sex (reference: male)	0.65 (0.46–0.91)	0.011	0.59 (0.40–0.86)	0.006
Age (per year)	1.04 (1.02–1.05)	<0.001	1.05 (1.04–1.07)	<0.001
Child Pugh				
• A (reference)				
• B	1.92 (1.30–2.82)	0.001	1.54 (1.03–2.32)	0.037
• C	3.96 (2.54–6.16)	<0.001	4.29 (2.57–7.17)	<0.001
ALD (reference: non ALD)	1.65 (1.20–2.26)	0.002	0.81 (0.56–1.17)	0.264
Variceal bleeding at inclusion	1.62 (1.18–2.23)	0.003	1.37 (0.95–1.96)	0.089

* P-values were calculated by Fine and Grey

We found, however, no difference in cumulative incidence of death without LT according to size of esophageal varices (SHR 1.32 [0.95–1.83]) or presence of gastric varices (SHR 0.96 [0.64–1.43]).

In a multivariate model including these factors, risk of death without LT was still associated with female sex (SHR 0.59 [0.40–0.86]), age (SHR 1.05 [1.04–1.07] per year), Child Pugh class B (SHR 1.54 [1.03–2.32]) and Child Pugh class C (SHR 4.29 [2.57–7.17]). However, ALD and variceal bleeding at inclusion were not associated with risk of death without LT in the multivariate model (Table 3).

Separate calculations in patients with (n = 105) or without (n = 161) variceal bleeding at inclusion showed that reduced cumulative incidence of death without LT in females was restricted to patients presenting with variceal bleeding (S1 Fig). Accordingly, multivariate analyses confirmed that female sex was significantly associated with reduced risk of death without LT in patients presenting with variceal bleeding (SHR 0.47 [0.25–0.89]) but not in patients with no variceal bleeding at inclusion (SHR 0.65 [0.39–1.09]) (S2 Table).

On the other hand, separate multivariate analyses according to Child Pugh class A (n = 83) or Child Pugh class B/C (n = 183), confirmed an independent association between female sex and risk of death without LT in both Child Pugh A (SHR 0.42 [0.18–0.96]) and Child Pugh B/C patients (SHR 0.63 [0.42–0.96]) (S3 Table).

### Cumulative incidences and risk of LT

Thirty-seven patients (13.9%) received LT during follow-up. Considering death as a competing event, the five years cumulative incidence of LT was 0.13 (95% CI [0.09–0.17]). The cumulative incidence was lower in patients with ALD compared to patients with non-ALD (S2 Fig). After adjusting for age and gender, ALD patients had 66% reduced risk of LT compared to non-ALD patients (SHR 0.34; 95% CI [0.15–0.75], p = 0.007).

Our data did not reveal any further differences in cumulative incidences or risk of LT according to gender or ethnicity (Caucasian versus non-Caucasian).

### Causes of death

Causes of death could be assessed in 147 of 157 patients (93%) who died without LT during follow up (Table 4).

**Table 4:** Causes of death among 157 patients with liver cirrhosis and gastroesophageal varices who died without LT. Data are numbers (%).

Liver death—variceal bleeding 43 (27)	Liver failure and variceal bleeding	27 (17)
	Variceal bleeding without liver failure	16 (10)
Liver death–no variceal bleeding 59 (38)	Liver failure and infection	21 (13)
	Liver failure and non variceal bleeding	2 (1)
	Liver failure (no infection, no bleeding)	20 (13)
	HCC	16 (10)
Non liver death 45 (29)	Malignancy (not HCC)	14 (9)
	Infection without liver failure	17 (11)
	Cardiovascular disease	6 (4)
	Other non liver related causes	8 (6)
Unknown cause 10 (6)		10 (6)

Among these patients, 48% had liver failure at time of death and 29% died due to variceal bleeding. Altogether, 69% died due to liver disease. Mean age at death was significantly lower among those who died of variceal bleeding as compared to patients who died of other aspects of liver disease; mean (SD) were 60.9 (13.4) years and 66.0 (10.6) years, respectively, p = 0.036. While age at inclusion was a predictor of mortality due to liver disease other than variceal bleeding (SHR 1.04 per year; 95% CI [1.02–1.06], p<0.001) and non-liver related death (SHR 1.02 per year; 95% CI [1.00–1.05], p = 0.045) age was not associated with death due to variceal bleeding (SHR 1.01 per year; 95% CI [0.98–1.03], p = 0.54).

**Fig 3:**
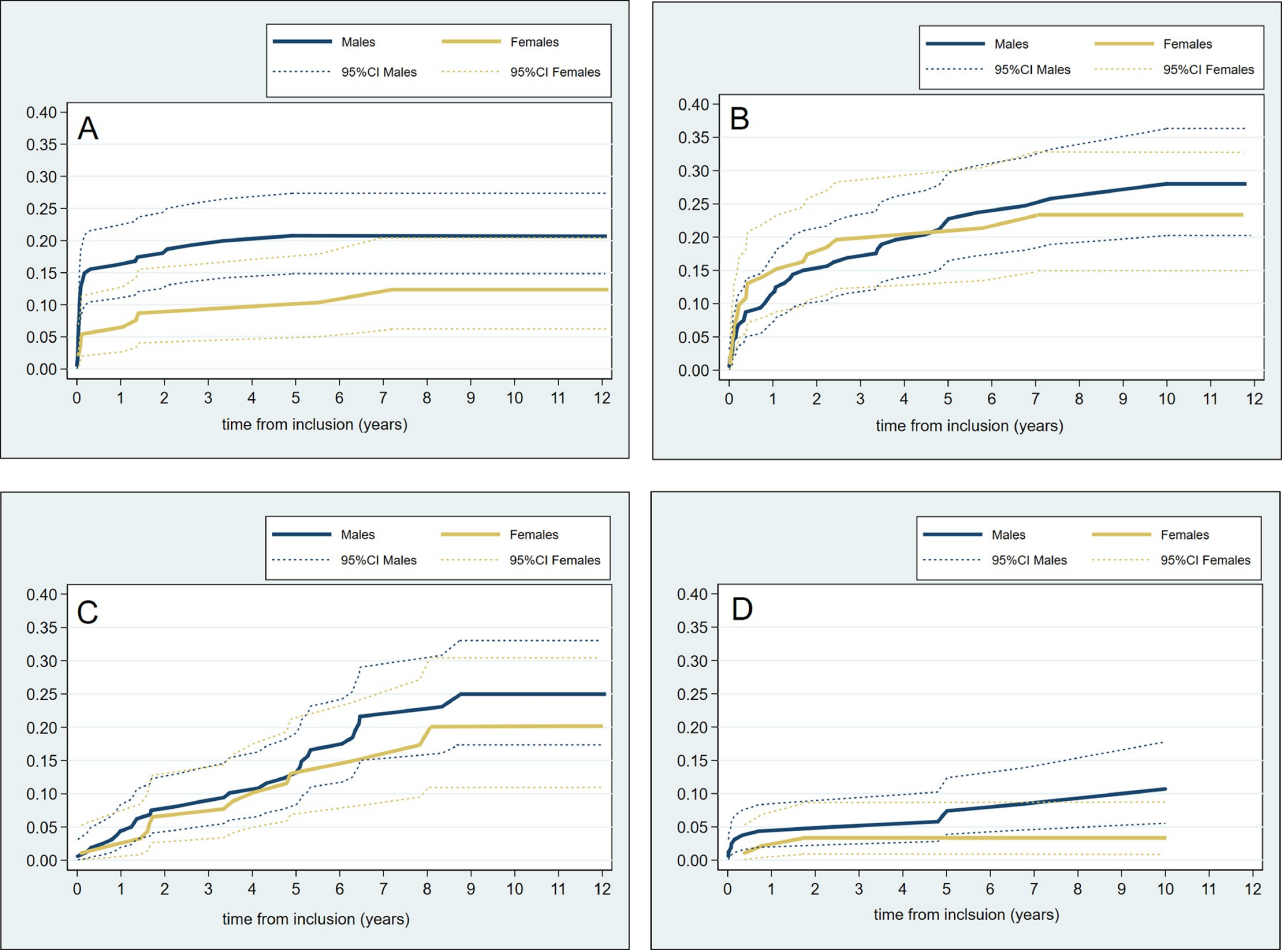
Cumulative incidences of specific causes of death without LT according to gender in 147 patients with known cause of death. The incidence curves were constructed with the Fine and Grey approach treating LT and all other causes of death as competing events. Death due to (A) variceal bleeding, (B) liver disease other than variceal bleeding, (C) non-liver disease and (D) hepatocellular carcinoma.

In order to explore our findings of reduced cumulative incidence of death without LT in women, we calculated cumulative incidences of cause specific death stratified by gender (Fig 3), and found lower cumulative incidences of both death due to variceal bleeding and death due to HCC in women as compared to men. In line with these findings, women had significantly reduced risk of death due to variceal bleeding (SHR 0.47; 95% CI [0.24–0.94], p = 0.033 and a trend towards reduced risk of death due to HCC (HR 0.32; 95% CI [0.092–1.10], p = 0.071), both models were adjusted for age. After adjusting for both age and Child Pugh class, there was still a trend towards reduced risk of death due to variceal bleeding in women (SHR 0.53; 95% CI [0.26–1.06], p = 0.071). On the other hand, gender was not associated with risk of liver related death other than variceal bleeding nor with non-liver related death.

No differences in cause-specific death were seen when patients with ALD were compared to non-ALD patients.

### Predictors of first variceal bleeding

Among 151 patients with neither history of variceal bleeding nor variceal bleeding at inclusion, the one- and five years cumulative incidence of first variceal bleeding were 0.06 (95% CI [0.03–0.11]) and 0.13 (95% CI [0.07–0.21]), respectively. No differences in crude cumulative incidence of first variceal bleeding were seen according to gender, age, Child Pugh class, grade of esophageal varices or presence of gastric varices. However, cumulative incidence was higher in patients with high alcohol consumption (i.e. ALD or viral hepatitis with high alcohol consumption, n = 70) compared to the remaining patients (S3 Fig). Patients in this group had increased risk of variceal bleeding both in univariate analyses (SHR = 7.73; 95% CI [1.71–34.90]), p = 0.008), and after adjusting for gender, age, Child Pugh class and grade of esophageal varices (SHR 13.91; 95% CI [2.51–76.98], p = 0.003).

### Predictors of first variceal re-bleeding after inclusion

Among 115 patients that either presented with variceal bleeding or had a history of variceal bleeding prior to inclusion, the one- and five years cumulative incidence of variceal re-bleeding were 0.13 (95% CI [0.06–0.22]) and 0.32 (95% CI [0.18–0.46]), respectively. No differences in cumulative incidence or risk of variceal re-bleeding was seen according to gender, age, Child Pugh class, alcohol consumption, grade of esophageal varices or presence of gastric varices.

### Incidence rates of gastroesophageal varices due to liver cirrhosis

Estimated annual incidence rates of gastroesophageal varices due to liver cirrhosis for non- African and non-Asian patients were stable throughout the study period, averaging 5.8 new cases per 100.000. Among African and Asian immigrants the average yearly incidence rate was 7.8 per 100.000. Combining these estimates, we calculated an age- and gender- weighted overall yearly incidence rate of 5.9 new cases per 100.000 in Norway.

## Discussion

The main finding in the present retrospective study of 266 cirrhotic patients with gastroesophageal varices was reduced cumulative incidence and reduced risk of death without LT in women, possibly explained by reduced mortality due to variceal bleeding and HCC.

Female sex was associated with significant reduction in risk of death without LT after adjustment for age, Child Pugh class, etiology and mode of presentation (variceal bleeding or not). Importantly, the improved prognosis in females was seen both in compensated and decompensated disease. Little attention has been paid to the role of gender regarding survival in patients with cirrhosis. As reviewed by D’Amico [1], some reports have identified gender as a predictor of improved survival, while other studies have found no such effect. However, during the last decade some new reports in line with our findings have emerged. In a Taiwanese cohort including 3409 patients with non-alcoholic cirrhosis, males had 20% increased mortality [7]. Among 752 patients with cirrhosis due to ALD, NAFLD or HCV infection, 36% increased mortality was observed in males [8]. In a Swedish study of 1317 patients with cirrhosis, mortality was reduced by 25% in women [9]. On the other hand, in a study exploring safety of carvedilol in 264 cirrhotic patients with ascites, gender had no effect on survival [10]. The differences reported may relate to different study populations with regard to etiologies of cirrhosis, degree of hepatic decompensation and length of follow-up.

To the best of our knowledge, no studies have explored possible relationships between survival, different causes of death and gender in patients with cirrhosis. By performing a comprehensive evaluation of causes of death our findings may shed light on the observed gender difference in survival. Notably, the protective effect of female gender was stronger in cases with variceal bleeding at inclusion, indicating that the gender difference in survival may relate to variceal bleeding. In fact, when we explored the risk of dying of variceal bleeding, female sex was associated with reduced risk after adjusting for age, and a trend towards reduced risk in woman were still seen when we adjusted for both age and Child Pugh class. Importantly, the gender effect on mortality due to variceal bleeding could not be explained by a difference in bleeding episodes as the cumulative incidences of both first variceal bleeding and variceal re-bleeding were the same in both groups. Our results are in accordance with a recently published Italian study which found in-hospital mortality of acute variceal bleeding to be approximately ten percent reduced in females, in particular among patients with non-alcoholic liver disease [11].

In our study a substantial fraction of patients presenting with variceal bleeding died within a short time frame. Interestingly, in two large studies exploring factors associated with in-hospital mortality among patients with lower gastrointestinal bleeding, female sex was independently associated with reduced mortality [12, 13]. Studies have indicated that males are more prone to immune-depression after trauma and haemorrhage [14]. As sepsis is a well-known complication of variceal bleeding, this gender-specific immunological dimorphism could explain some of the increased mortality due to variceal bleeding in males.

One could speculate whether there is a gender difference related to patient delay before seeking medical help for symptoms of gastrointestinal bleeding. Unfortunately, this question could not be assessed in the present study due to the retrospective design. A recent study exploring time to presentation after onset of symptoms of gastrointestinal bleeding in almost 3000 patients found no gender difference in proportions attending hospital within six hours, however, more than 48 hours delay (20% of the patients) was significantly more often observed among men [15].

Another possible explanation for reduced mortality in women is the increased risk of HCC among men [16]. As expected, the mortality due to HCC was significantly higher in males in our study.

As the reduced survival in men may relate to higher incidence of death due to HCC and variceal bleeding, primary prophylaxis of variceal bleeding and surveillance of HCC may be of particular importance in this group, though still indicated in both men and women.

It is not unlikely that the gender effect depends upon type of complication of the liver disease, highlighting the need to assess cause specific death in order to understand the role of gender on prognosis in cirrhosis. Interestingly, mortality in cirrhotic patients with bacterial peritonitis was actually found to be increased in women as compared to men in a recently published study by Niu et al [17].

Whether prognosis is different in patients with ALD than in other groups is still under debate. A Swedish study reported increased mortality among patients with ALD compared to HCV patients without high alcohol consumption [9]. Likewise, Marot et al found reduced long term survival in ALD patients who continued drinking [8], in abstainers, however, survival was similar in patients with ALD, NAFLD and HCV infection, as also reported in a Japanese study comparing survival in patients with ALD and HCV infection [18]. On the other hand, a trend towards improved survival was observed in ALD patients as compared to HCV-patients in a Spanish study, however, this study was conducted before the era of direct acting antiviral HCV treatment [19]. Whether etiology affects survival is a complex question as factors such as patient selection, length of follow-up, degree of decompensation, viral eradication and alcohol abstinence all influence survival. In our study, due to limited number of subjects, we compared ALD patients with the remaining etiologies, which represents a rather heterogeneous group. Although ALD was associated with poorer prognosis at univariate analysis, we found no difference in cumulative incidence or risk of death without LT according to etiology when controlling for other factors. Interestingly, however, among patients with no previous history of variceal bleeding, alcohol consumption was a strong predictor of first bleeding episode, confirming results from a Taiwanese study [20].

Gastroesophageal varices indicate an advanced state of cirrhosis and half of the patients died without LT within five years after inclusion. In accordance with other studies [8, 9], about two of three patients in our study died as a consequence of the liver disease, and a substantial fraction of the patients died due to variceal bleeding. Notably, in the subgroup of 151 patients with no variceal bleeding before or at inclusion, 7.9% died due to variceal bleeding during follow up, despite varices being diagnosed previously. This finding emphasizes the importance of regular follow-up and implementation of primary prophylaxis whenever indicated, in particular in patients with high alcohol consumption given the increased risk of variceal bleeding in this group.

Epidemiological studies based on data registered in administrative systems are inherently imprecise as coding of diagnoses may be incomplete or erroneous. A strength of our study relates to the strictness in which causes of death could be assessed by studying patient records in the majority of cases, restricting the use of data from the Norwegian Death Registry to a minimum. Importantly, the cumulative incidences of various causes of death were assessed by competing risk analyses to avoid the overestimation typically seen with inverse Kaplan-Meier plots [21]. Furthermore, in Norway, the same treatment is offered regardless of socio-economic status, and access to hospital is free of cost for all patients, enhancing standardized follow up for all included patients. Although data collection was done retrospectively, very few clinical and biochemical variables were missing. The anticipated strong association between Child Pugh class and mortality [1] may confirm the high quality of the data collection. The calculated annual incidence rate of 5.9 new cases with gastroesophageal varices because of cirrhosis per 100.000 per year fits well with a previously found annual incidence rate of cirrhosis in Norway of 13.4 new cases per 100.000 per year [22], indicating that the present cohort represent a complete and unbiased sample from an unselected population of cirrhotic patients in Norway.

There are several limitations. Due to the retrospective design we were not able to obtain reliable data regarding alcohol abstinence, which is known to improve prognosis [23], nor was presence of comorbid conditions such as diabetes and obesity captured. Furthermore, by combining patients from OUS catchment area with patients living outside this area, we introduced a more heterogeneous population. The latter group was biased towards more serious conditions as many of them were referred because they needed treatment at a tertiary centre, in particular in relation to variceal bleeding. Higher mortality in this group could be explained by higher Child Pugh state, we therefore included them in the survival analyses. As the main objective was to assess possible associations between selected variables and the outcomes, we assume that the increased heterogeneity of our sample did not introduce bias.

In conclusion, we report decreased mortality in female patients with gastroesophageal varices due to cirrhosis independent of Child Pugh state, and hypothesize this to be caused by lower mortality due to variceal bleeding and HCC.

## Supporting information

S1 Fig Cumulative incidences of death according to gender among patients presenting with or without variceal bleeding. (PPTX)

S2 Fig Cumulative incidences of liver transplantation according to etiology. (PPTX)

S3 Fig Cumulative incidence of first variceal bleeding according to alcohol consumption. (PPTX)

S1 Table Protocol for assessment of cause of death. (DOCX)

S2 Table Competing risk regression to explore factors associated with risk of death without LT according to whether variceal bleeding was present or not at inclusion. (DOCX)

S3 Table Competing risk regression to explore factors associated with risk of death without LT according to Child Pugh class. (DOCX)
