# Supplementary material for: Effect of gender on mortality and causes of death in cirrhotic patients with gastroesophageal varices. A retrospective study in Norway
Source: PLoS One. 2020 Mar 12;15(3):e0230263. doi: 10.1371/journal.pone.0230263 (PMC7067466; doi:10.1371/journal.pone.0230263)
Supplement: S1 Fig — (PPTX) [file pone.0230263.s001.pptx]

## Slide 1
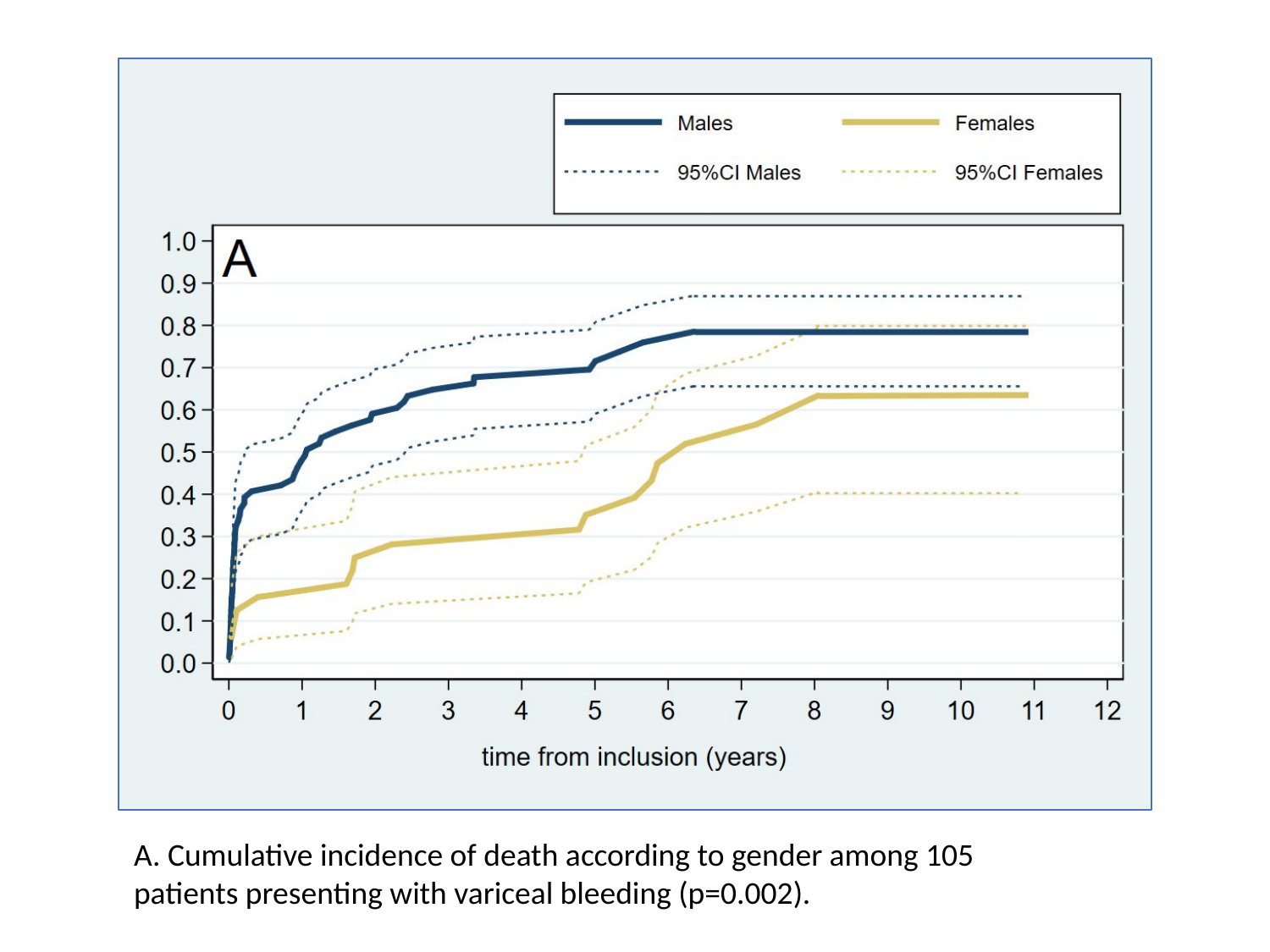

A. Cumulative incidence of death according to gender among 105 patients presenting with variceal bleeding (p=0.002).

## Slide 2
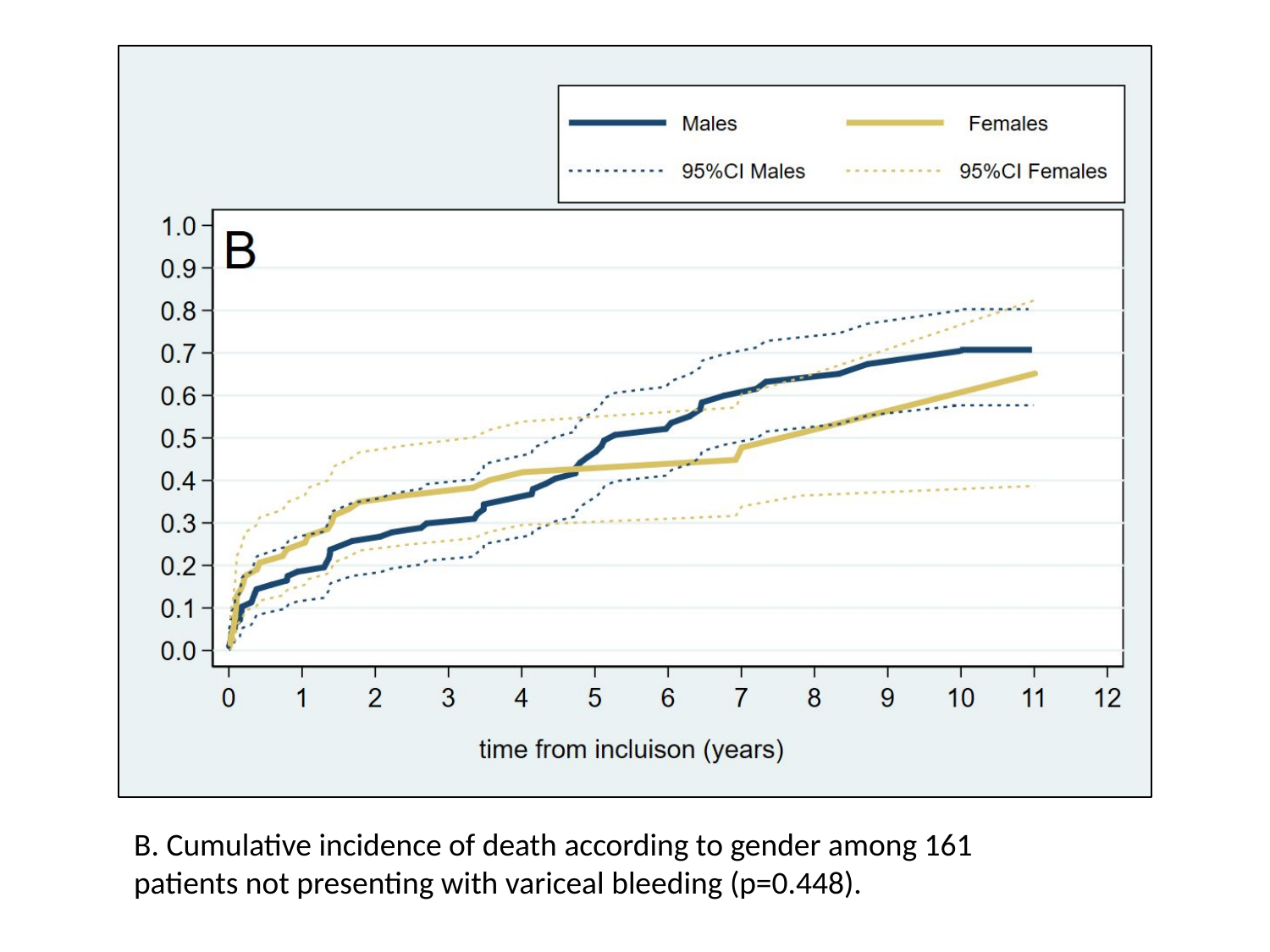

B. Cumulative incidence of death according to gender among 161 patients not presenting with variceal bleeding (p=0.448).
