# Supplementary material for: Effect of gender on mortality and causes of death in cirrhotic patients with gastroesophageal varices. A retrospective study in Norway
Source: PLoS One. 2020 Mar 12;15(3):e0230263. doi: 10.1371/journal.pone.0230263 (PMC7067466; doi:10.1371/journal.pone.0230263)
Supplement: S2 Fig — (PPTX) [file pone.0230263.s002.pptx]

## Slide 1
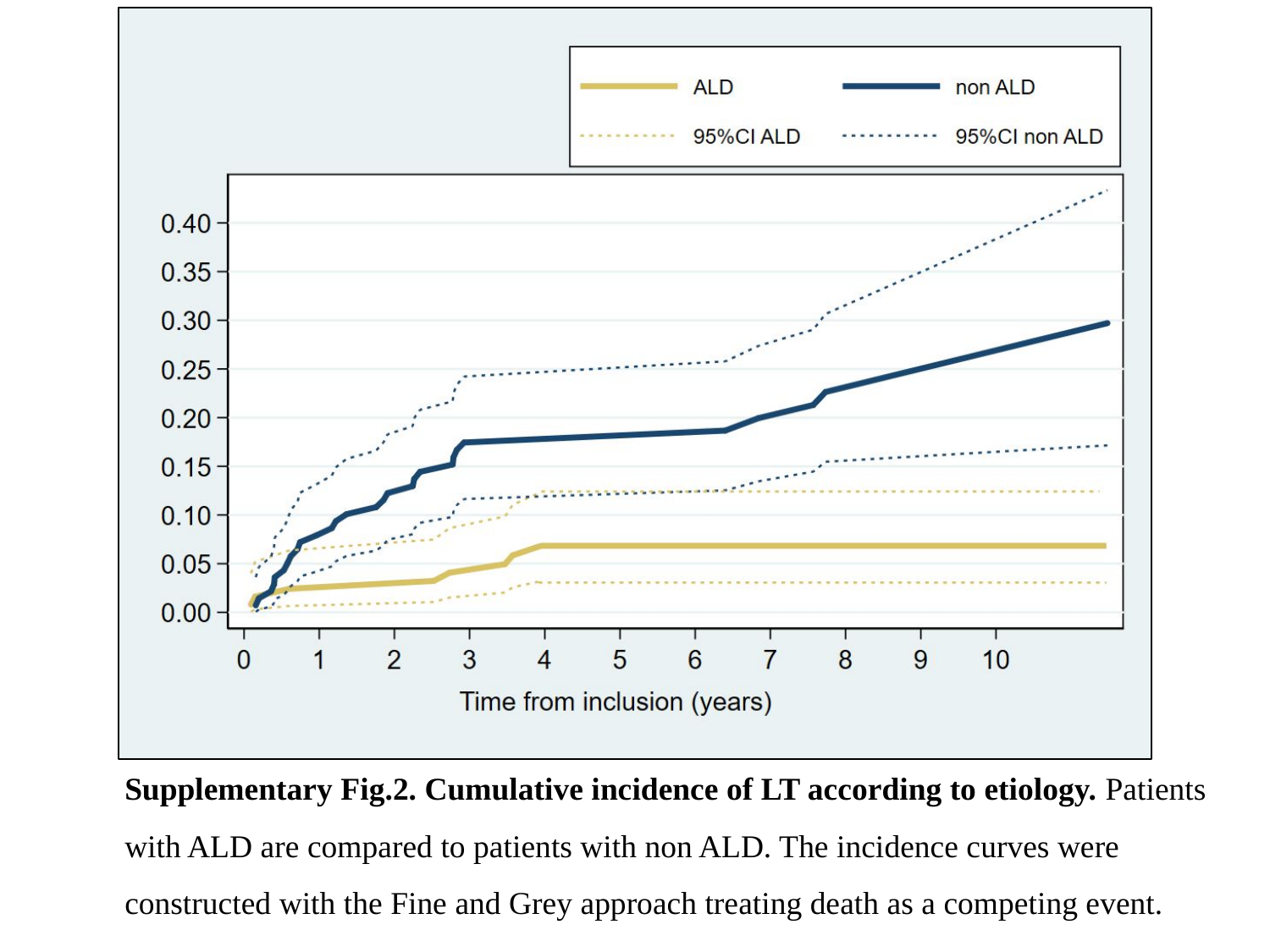

Supplementary Fig.2. Cumulative incidence of LT according to etiology. Patients with ALD are compared to patients with non ALD. The incidence curves were constructed with the Fine and Grey approach treating death as a competing event.
