# Supplementary material for: Effect of gender on mortality and causes of death in cirrhotic patients with gastroesophageal varices. A retrospective study in Norway
Source: PLoS One. 2020 Mar 12;15(3):e0230263. doi: 10.1371/journal.pone.0230263 (PMC7067466; doi:10.1371/journal.pone.0230263)
Supplement: S3 Fig — (PPTX) [file pone.0230263.s003.pptx]

## Slide 1
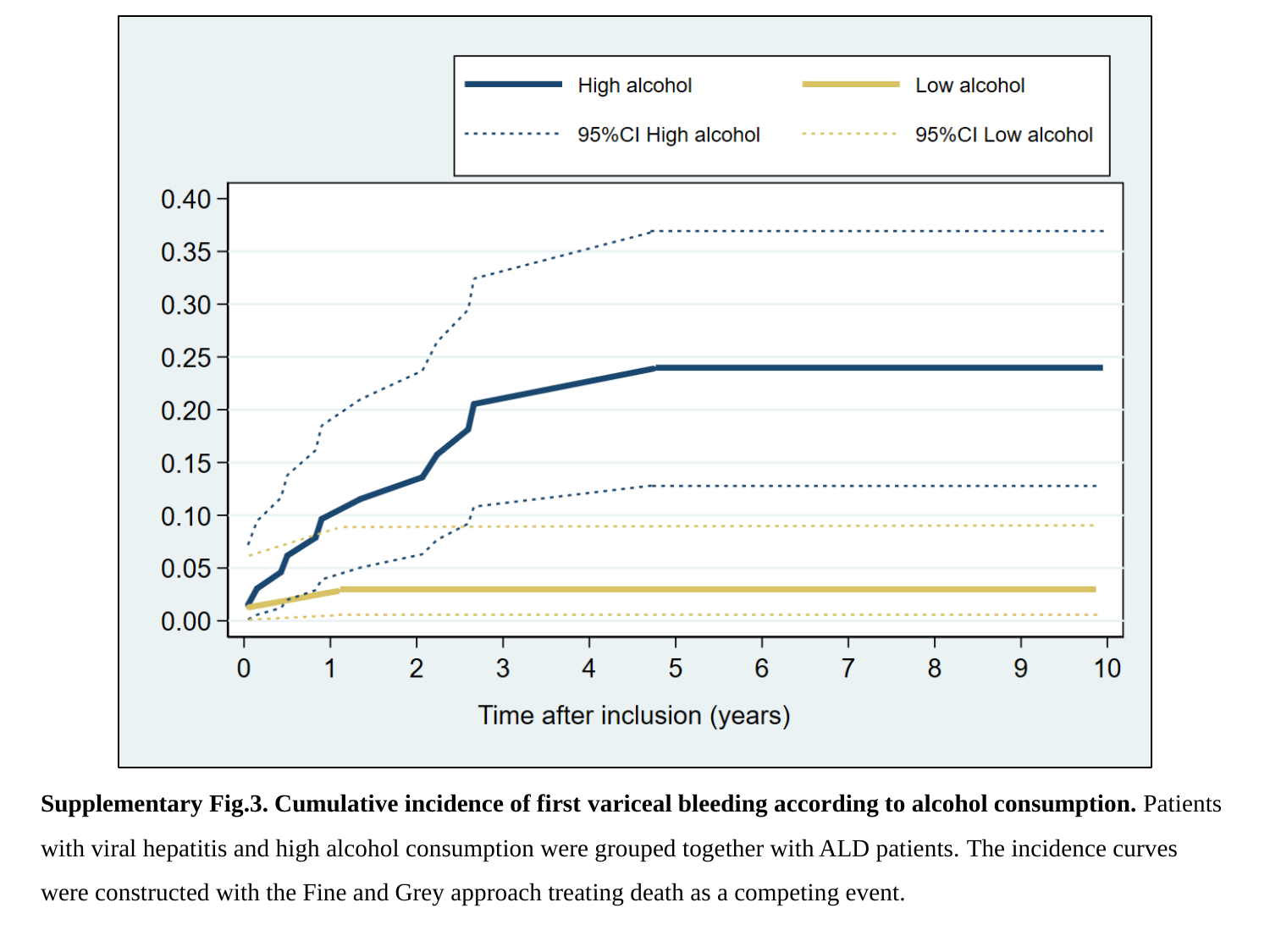

Supplementary Fig.3. Cumulative incidence of first variceal bleeding according to alcohol consumption. Patients with viral hepatitis and high alcohol consumption were grouped together with ALD patients. The incidence curves were constructed with the Fine and Grey approach treating death as a competing event.
