# Supplementary material for: Effect of gender on mortality and causes of death in cirrhotic patients with gastroesophageal varices. A retrospective study in Norway
Source: PLoS One. 2020 Mar 12;15(3):e0230263. doi: 10.1371/journal.pone.0230263 (PMC7067466; doi:10.1371/journal.pone.0230263)
Supplement: S1 Table — (DOCX) [file pone.0230263.s004.docx]

**Protocol for assessment of cause of death**

Cause of death was classified as:

- Death **because of** liver disease
  1. Liver failure and infection
  2. Liver failure and variceal bleeding
  3. Liver failure and non variceal bleeding
  4. Liver failure (no infection, no bleeding)
  5. Variceal bleeding without liver failure
  6. HCC
- Death **not because of** liver disease
  1. Malignancy (not HCC)
  2. Infection without liver failure
  3. Cardiovascular disease
  4. Other non liver related causes
- **Unknown** cause of death

Patient records were studied rigorously to identify the main disease or disease complication(s) causing death. In accordance with definitions suggested by EASL CLIF Consortium in the context of acute-on-chronic liver failure (1), biochemical criteria for liver failure were defined as either INR > 2.5 or serum bilirubin > 200 μmol/L. Liver failure was also defined as the cause of death in patients with hepatorenal syndrome, encephalopathy or cachexia when no other non liver related cause was identified. Furthermore, spontaneous bacterial peritonitis was defined as liver failure and infection even if biochemical criteria for liver failure were not fulfilled.

When two or more possible causes were present, cause of death was defined as the most important cause during the last weeks prior to death that initiated a downhill cause leading to death. In patients presenting with sepsis and multiorgan failure that developed variceal bleeding at a later stage during the hospital stay, cause of death was defined as infection (with or without liver failure). On the other hand, in patients presenting with variceal bleeding, cause of death was defined as variceal bleeding (with or without liver failure) even if severe infection developed at a later stage during the hospital stay.

Hepatocellular carcinoma (HCC) was defined as the cause of death in all patients with advanced HCC, except in cases were variceal bleeding clearly contributed to the death.

For patients not dying in hospital (or shortly thereafter), cause of death was based on data from the Norwegian Cause of Death Registry.

**References**

1. Arroyo V, Moreau R, Jalan R, Gines P. Acute-on-chronic liver failure: A new syndrome that will re-classify cirrhosis. Journal of hepatology. 2015;62(1 Suppl):S131-43.
