# Supplementary material for: Effect of gender on mortality and causes of death in cirrhotic patients with gastroesophageal varices. A retrospective study in Norway
Source: PLoS One. 2020 Mar 12;15(3):e0230263. doi: 10.1371/journal.pone.0230263 (PMC7067466; doi:10.1371/journal.pone.0230263)
Supplement: S2 Table — (DOCX) [file pone.0230263.s005.docx]

**Supplementary Table 2.** Univariate and multivariate competing risk regression to explore factors associated with risk of death without LT according to wether variceal bleeding was present or not at inclusion.

|  | **Univariate** | | **Multivariate** | |
| --- | --- | --- | --- | --- |
|  | SHR (95% CI) | *p-value** | SHR (95% CI) | *p-value** |
| **No bleeding at inclusion (n=161)** | |  |  |  |
| Female sex (reference: male) | 0.84 (0.53-1.32) | 0.448 | 0.65 (0.39-1.09) | 0.099 |
| Age (per year) | 1.05 (1.03-1.07) | <0.001 | 1.06 (1.04-1.08) | <0.001 |
| ALD (reference: not ALD) | 1.56 (1.04-2.35) | 0.034 | 0.75 (0.47-1.21) | 0.235 |
| Child Pugh   - A (reference) - B - C | 1.93 (1.19-3.12)  4.46 (2.45-8.10) | 0.007  <0.001 | 1.49 (0.91-2.45)  4.89 (2.56-9.37) | 0.114  <0.001 |
| **Bleeding at inclusion (n=105)** | |  |  |  |
| Female sex (reference: male) | 0.45 (0.27-0.74) | 0.002 | 0.47 (0.25-0.89) | 0.019 |
| Age (per year) | 1.02 (1.0 – 1.04) | 0.056 | 1.04 (1.02-1.06) | 0.001 |
| ALD (reference: not ALD) | 1.54 (0.92-2.55) | 0.098 | 0.76 (0.40-1.46) | 0.417 |
| Child Pugh   - A (reference) - B - C | 1.46 (0.69-3.05)  2.64 (1.21-5.75) | 0.319  0.015 | 1.60 (0.70-3.68)  3.59 (1.35-9.58) | 0.266  0.011 |

* P-values are calculated by Fine and Grey
