# Supplementary material for: Effect of gender on mortality and causes of death in cirrhotic patients with gastroesophageal varices. A retrospective study in Norway
Source: PLoS One. 2020 Mar 12;15(3):e0230263. doi: 10.1371/journal.pone.0230263 (PMC7067466; doi:10.1371/journal.pone.0230263)
Supplement: S3 Table — (DOCX) [file pone.0230263.s006.docx]

**Supplementary Table 3.** Univariate and multivariate competing risk regression to explore factors associated with risk of death without LT according to Child Pugh class.

|  | **Univariate** | | **Multivariate** | |
| --- | --- | --- | --- | --- |
|  | SHR (95% CI) | *p-value** | SHR (95% CI) | *p-value** |
| **Child Pugh A (n=83)** |  |  |  |  |
| Female sex (reference: male) | 0.71 (0.36-1.42) | 0.336 | 0.42 (0.18-0.96) | 0.039 |
| Age (per year) | 1.05 (1.02-1.08) | 0.002 | 1.06 (1.02-1.10) | 0.001 |
| ALD (reference: not ALD) | 2.83 (1.50-5.38) | 0.001 | 1.95 (0.93-4.11) | 0.078 |
| Variceal bleeding at inclusion | 1.76 (0.74-4.19) | 0.199 | 1.82 (0.71-4.64) | 0.212 |
| **Child Pugh B or C (n=183)** |  |  |  |  |
| Female sex (reference: male) | 0.69 (0.47-1.02) | 0.066 | 0.63 (0.42-0.96) | 0.032 |
| Age (per year) | 1.03 (1.02-1.05) | <0.001 | 1.04 (1.02-1.05) | <0.001 |
| ALD (reference: not ALD) | 1.04 (0.73-1.49) | 0.824 | 0.86 (0.59-1.25) | 0.428 |
| Variceal bleeding at inclusion | 1.21 (0.86-1.72) | 0.277 | 1.42 (0.99-2.03) | 0.058 |

* P-values are calculated by Fine and Grey
